# Burnei’s “double X" internal fixation technique for supracondylar humerus fractures in children: indications, technique, advantages and alternative interventions: Study and Research Group in Pediatric Orthopaedics-2012

**Published:** 2013-06-25

**Authors:** I Georgescu, S Gavriliu, A Pârvan, A Martiniuc, E Japie, R Ghiță, I Drăghici, S Hamei, I Ţiripa, T El Nayef, D Dan

**Affiliations:** * "Maria Sklodowska Curie" Emergency Hospital for Children, Bucharest, Romania; ** "Carol Davila" University of Medicine and Pharmacy, Bucharest, Romania; *** "Floreasca" Emergency Hospital, Bucharest, Romania

**Keywords:** supracondylar humeral fracture, percutaneous pinning, open reduction, double X osteosynthesis

## Abstract

Background. The Study and Research Group in Pediatric Orthopedics-2012 initated this retrospective study due to the fact that in Romania and in other countries, the numerous procedures do not ensure the physicians a definite point of view related to the therapeutic criteria in the treatment of supracondylar fractures. That is why the number of complications and their severity brought into notice these existent deficiencies. In order to correct some of these complications, cubitus varus or valgus, Prof. Al. Pesamosca communicated a paper called "Personal procedure in the treatment of posttraumatic cubitus varus" at the County Conference from Bacău, in June 24, 1978. This procedure has next been made popular by Prof. Gh. Burnei and his coworkers by operating patients with cubitus varus or valgus due to supracondylar humeral fractures and by presenting papers related to the subject at the national and international congresses. The latest paper regarding this problem has been presented at the 29th Annual Meeting of the European Pediatric Orthopedic Society in Zagreb, Croatia, April 7-10, 2010, being titled “Distal humeral Z-osteotomy for posttraumatic cubitus varus or valgus", having as authors Gh. Burnei, Ileana Georgescu, Ştefan Gavriliu, Costel Vlad and Daniela Dan.

As members of this group, based on the performed studies, we wish to make popular this type of osteosynthesis, which ensures a tight fixation, avoids complications and allows a rapid postoperative activity.

Introduction. The acknowledged treatment for these types of fractures is the orthopedic one and it must be accomplished as soon as possible, in the first 6 hours, by reduction and cast immobilization or by closed or open reduction and fixation, using one of the several methods (Judet, Boehler, Kapandji, San Antonio, San Diego, Burnei’s double X technique). The exposed treatment is indicated in irreducible supracondylar humeral fractures, in reducible, but unstable type, in polytraumatized patients with supracondylar fractures, in supracondylar fractures with vascular injury, in late presenting fractures, in case of loss of reduction under cast immobilization or in case of surgery with other types of fixation that is deteriorated. We have been using Burnei’s osteosynthesis for about 10 years.

Aim. This paper aims to present the operative technique, its results and advantages.

Materials and methods. 56 cases were treated with Burnei’s "double X" osteosynthesis in "Alexandru Pesamosca" Surgery Clinics, from 2001 to 2011. We used the Kocher approach and the aim of surgery was to obtain a fixation that does not require cast immobilization and that allows motion 24 hours after the surgery. The wires placed in "double X" must not occupy the olecranon fossa. The reduction must be anatomical and the olecranon fossa free. Flexion and extension of the elbow must be in normal range after surgery without crackles or limitations.

This surgery was performed on patients with:

• Loss of reduction after 10 days with cast immobilization;

• Surgery with other types of fixation, deteriorated;

• Polytraumatized patients with supracondylar fracture;

• After neglected or late presenting fractures, without the orthopedic reduction made in emergency;

• Fractures with edema and blistering.

Results and complications. The patients’ ages ranged 3 to12 years old, the mean age for girls was 7,3 years and 6,8 for boys. The hospitalization ranged 3 to 7 days, the average period being of 5 days. The wires had been pulled out after 21 days. The total recovery of the flexion and extension motion of the elbow was, depending on the age, between 21 and 40 days with an average period of 30 days.

There were 5 cases of minor complications: in 3 cases the wires migrated outwards up to the 10th day and in 2 cases the wires were found in the olecranon fossa. The CT exam highlighted the impingement effect and the wire that passed through the olecranon fossa had to be removed between the 7th and the 9th day. No reported cases of cubitus varus or valgus were reported.

Conclusion. The Burnei’s "double X" osteosynthesis does not require cast immobilization. In oblique fractures, the stability is more difficult to obtain and by using other methods, elbow stiffness or ulnar nerve palsy may appear. The Burnei’s "double X" osteosynthesis ensures stability of these types of fractures and avoids complications. This technique allows early motion after surgery and, in case of polytrauma, ensures comfort both to the patient and the physician, allowing repetitive examinations, preferential positions or the nursing of the extensive skin lesions.

## Introduction

The Study and Research Group in Pediatric Orthopedics-2012 initated this retrospective study due to the fact that in Romania and in other countries, the numerous therapeutical procedures do not ensure the physicians a definite point of view related to the therapeutic criteria in the treatment of supracondylar fractures. That is why the number of complications and their severity brought into notice these existent deficiencies. In order to correct some of these complications, cubitus varus or valgus, Prof. Al. Pesamosca communicated a paper called “Personal procedure in the treatment of posttraumatic cubitus varus" at the County Conference from Bacău, in June 24, 1978. This procedure has next been made popular by Prof. Gh. Burnei and his coworkers by operating patients with cubitus varus or valgus due to supracondylar humeral fractures and by presenting papers related to the subject at national and international congresses. The latest paper regarding this problem has been presented at the 29th Annual Meeting of the European Pediatric Orthopedic Society in Zagreb, Croatia, April 7-10, 2010, being titled “Distal humeral Z-osteotomy for posttraumatic cubitus varus or valgus", having as authors Gh. Burnei, Ileana Georgescu, Ştefan Gavriliu, Costel Vlad and Daniela Dan.

 As members of this group, based on the performed studies, we wish to popularize this type of osteosynthesis, which ensures a tight fixation, avoids complications and allows a rapid postoperative activity.

 Supracondylar humerus fracture is ranked 3rd in frequency in the traumatic pathology of children [**1**]. Also taking into account its potential complications, varus and valgus deformities, excessive impinging callus and even elbow osteoarthritis [**2**], this makes supracondylar elbow fracture an important subject of research. 

 The treatment of displaced fractures can be achieved in a number of ways, starting with closed reduction and immobilization [**3,4**], and including open reduction and internal fixation with wires [**5**], screws and/or special plates.

In this paper, we present the technique of internal wire fixation of supracondylar humerus fractures by using four wires in a "double X" configuration. This technique exhibits numerous advantages.

## Materials and method

 56 cases were treated with Burnei’s "double X" osteosynthesis in the “Alexandru Pesamosca" Surgery Clinics, from 2001 to 2011. We used the Kocher approach and the aim of surgery was to obtain a fixation that does not require cast immobilization and that allows motion in 24 hours after surgery. 

 The "double X" osteosynthesis can be used for all displaced supracondylar humerus fractures, either by percutaneous pinning under image intensifier control, or by using an open approach for cases in which the fragments cannot be reduced and/or stabilized by using the closed method. 

 Technique for minimally invasive percutaneous pinning 

 Indications: displaced supracondylar fractures that are reducible and stable afterwards.

**Fig. 1:**
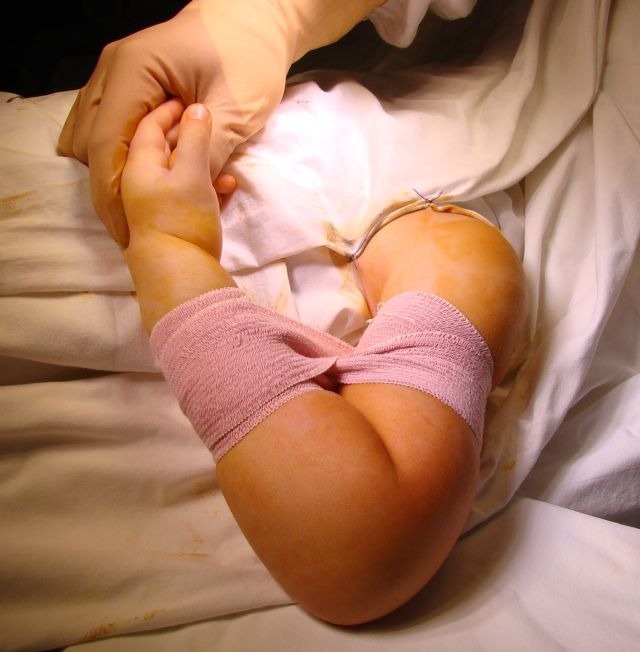
During surgery, reduction is maintained by using a bandage wrapped around the arm and forearm in a circle or "figure 8" shape

 The fractured limb is disinfected, draped and the fracture is reduced. The fracture is stabilized by using a “figure 8" or a circular bandage (**Fig. 1**).

 The first wire for each column of the distal humeral metaphysis is driven under X-ray control along the edge opposite to the olecranon fossa. Each wire must be introduced obliquely, in order to penetrate the opposite cortex. 

**Fig. 2:**
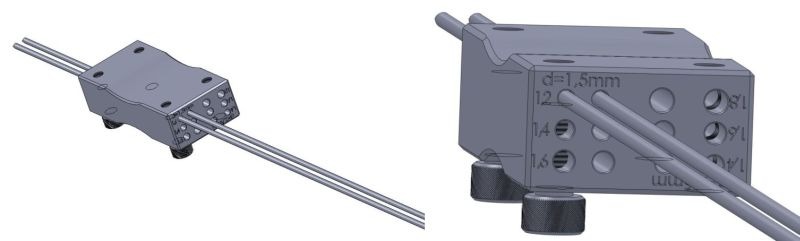
Guide for the parallel insertion of Kirschner wires that can accommodate multiple wire sizes and spacing, for bones of various sizes. The device has been developed and produced in cooperation with The National Institute of Research and Development in Mechatronics and Measurement Technique (INCMDMTM).

 In order for the wires to be parallel in the coronal plane, a guiding device for the parallel placement of Kirschner wires should be used, sizes 1.2, 1.4, 1.6 and 1.8 mm (**Fig. 2**). 

 The guide is placed on the first correctly placed wire and the second one is driven parallel to the first, under X-ray control in order to check that it has penetrated the opposite cortex, which adds a great deal of stability to the fixation.

**Fig. 3:**
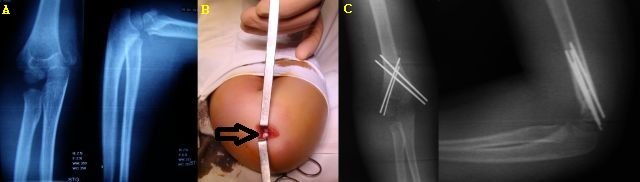
Supracondylar humeral fracture that was reduced and stabilized by using a minimally invasive technique. B. The ulnar nerve is identified through a minimal incision, in order to avoid injuring it. C. Postoperative X-ray.

 For patients with significant elbow edema preventing the palpation of the depression between the medial epicondyle and the olecranon, the risk of ulnar nerve injury can be avoided by locating the medial epicondyle by using the blunt end of a 2 mm Kirschner wire, inserted through a 2–3 mm incision. For inexperienced surgeons and in patients in whom the medial epicondyle is hard to locate with a blunt wire as described previously, a minimal 2–3 cm open approach can be used to insert the medial column wires (**Fig. 3 A,B**). The ulnar nerve is identified and protected and the wires are safely driven in.

 The final radiological aspect of the wires resembles a doubled letter "X", with one "X" offset a few millimeters laterally (**Fig. 3 C**).

**Fig. 4:**
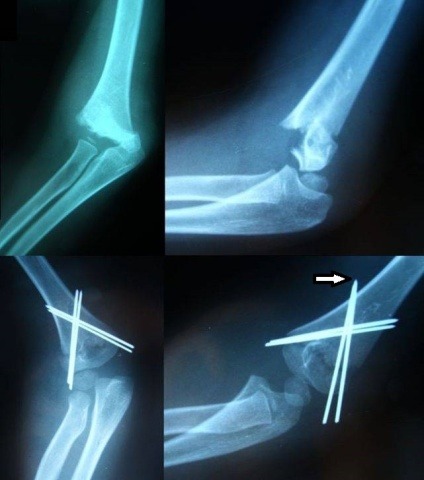
After fracture reduction and fixation, the wires are parallel in an oblique plane. Sometimes the wires are not parallel, but form a "V" shape pointing distally (arrow).

 If a wire guide is not used for the second wire of a column, it should be introduced parallel to the first in the coronal plane. The wires may be found to be parallel not in the coronal plane, but in an oblique one, after image intensifier control. Sometimes the wires are not parallel, forming a very asymmetric "V" shape pointing proximally or distally (**Fig. 4**).

 The use of a wire guide is preferred, which allows simple and efficient parallel placement of the wires. 

 Technique for open reduction

 Indications: supracondylar fractures that cannot be reduced or that are unstable after closed reduction, fractures in multiple trauma victims, undue delay of treatment, neglected fractures with edema and blisters, failures of orthopedic treatment (displacement under immobilization) or operated fractures with damaged fixation hardware.

**Fig. 5:**
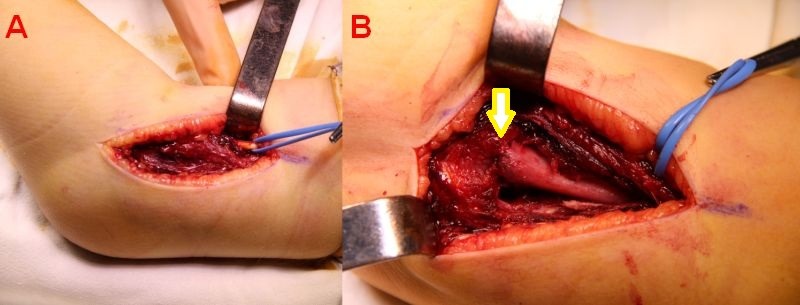
The approach: the septum between the extensor and flexor muscles is identified and retracted dorsally along with the extensor muscles; the flexor muscles are retracted ventrally. The radial nerve is identified and isolated for safety. B. Muscle entrapment between the fractured fragments.

**Fig. 6:**
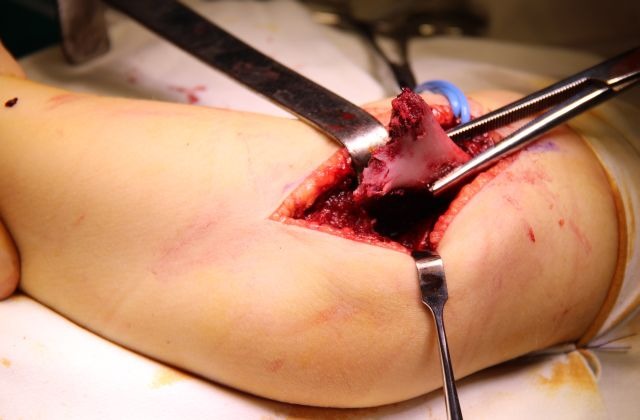
The proximal fragment is exposed outside the surgical wound. The columns of the humeral palette and their fractured ends are exposed.

 A Kocher incision and approach is used (**Fig. 5**). The periosteum is cut longitudinally on the external edge of the humerus and is stripped for 4–5 cm, enough to allow the exposure of the proximal fragment through the incision (**Fig. 6**).

**Fig. 7:**
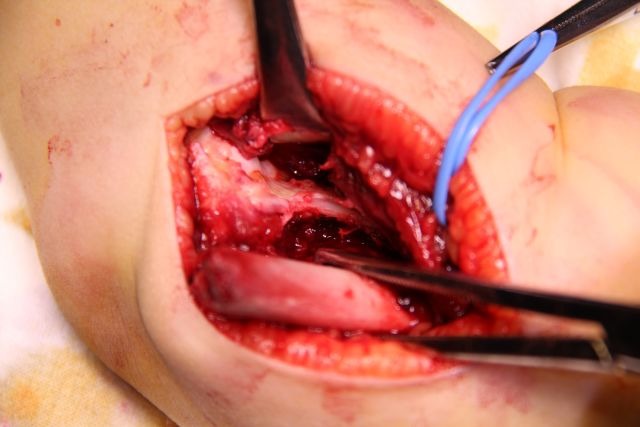
The ventral side of the distal fragment is exposed using a scalpel. The elbow joint and the medial edge of the fracture surface must be visualized.

 The distal fragment is exposed by using a scalpel, not a periosteal elevator, which can break off fragments of bone or damage the fractured surface. The opening of the articular capsule is mandatory in the case of articular fractures, in order to insure the anatomical reduction. The dissection must expose the medial edge of the humeral palette (**Fig. 7**).

**Fig. 8:**
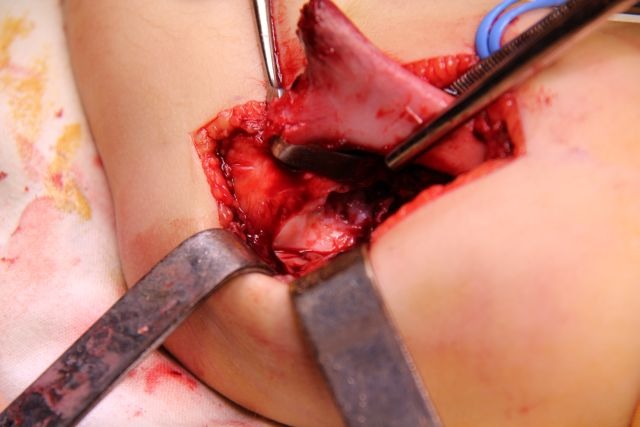
On the dorsal side of the distal fragment, the olecranon fossa must be identified

 Posteriorly, the olecranon fossa must be very accurately exposed (**Fig. 8**). The surgical wound must be carefully checked for small-comminuted fragments, which must be removed in order to avoid the formation of a voluminous callus, which may impinge on the olecranon and limit extension. 

**Fig. 9:**
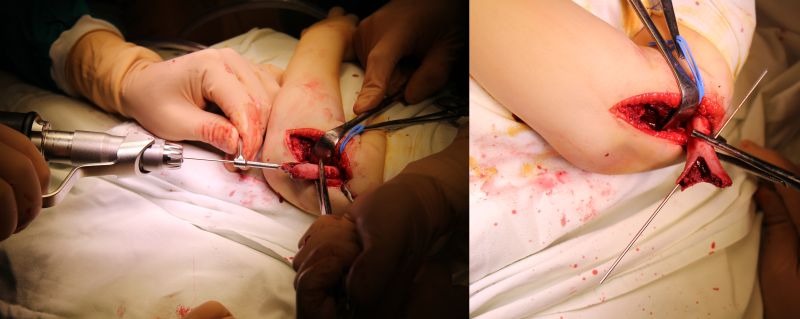
A wire is inserted along the outer wall of the medial pillar and is made to exit on the lateral side of the proximal fragment

 For the placement of the wires, the surgeon has several options:

 Option I:

 1. The proximal fragment is held in a bone clamp or a long elastic clamp and exposed through the incision. Two parallel wires are driven in an oblique angle through the fractured surface of the medial column, 1.5 – 3 mm apart (**Fig. 9-Fig. 11**). The steps are presented in drawing 1, scheme 1 and 2.

**Fig. 10:**
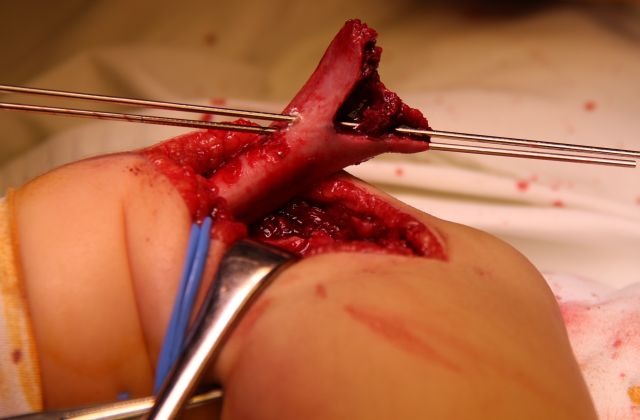
The second wire is placed parallel to the first one, which stabilizes firstly the comminuting fragment in this case

**Fig. 11:**
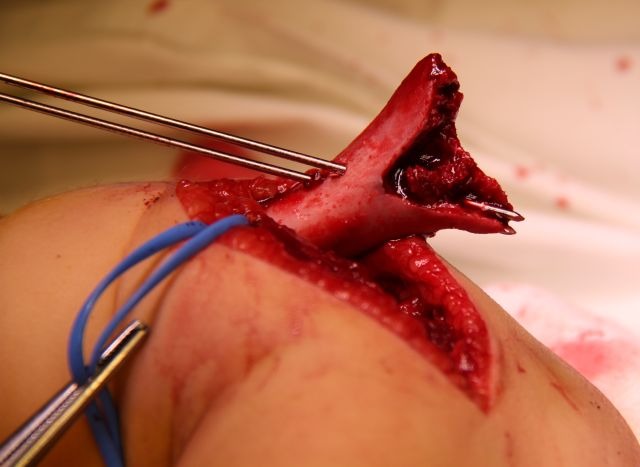
The points of the wires are placed flush to the fracture surface, in order to allow for reduction and subsequent fixation

**Fig. 12:**
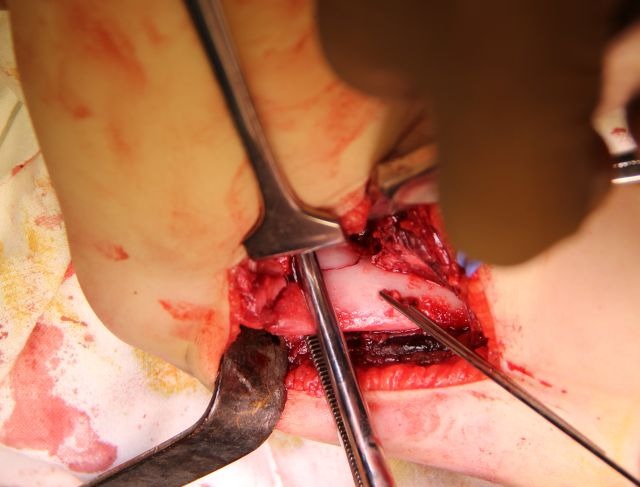
After the fracture is reduced, the wires are driven into the medial epicondyle, checking the extent of the penetration by palpation

**Fig. 13:**
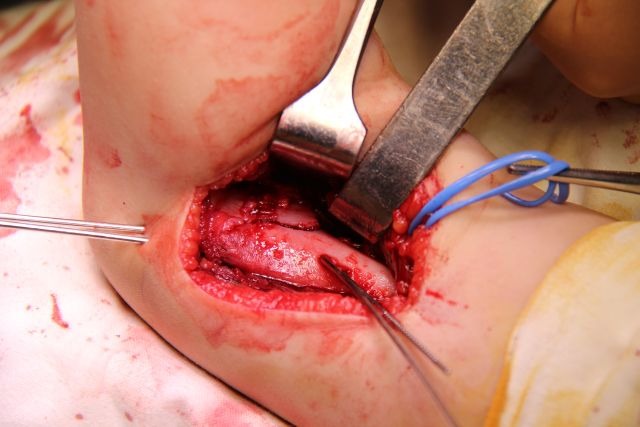
The other two wires are inserted into the lateral column under visual inspection, so that they penetrate the opposite cortex with a few millimeters

 2. If the fracture is above the olecranon fossa, the wires are placed toward the medial edge, in order for them to pass into the medial column when driven distally.

 3. After the wires are placed in the proximal fragment, the fracture is reduced with the elbow in flexion and the wires are driven in the distal fragment as it is shown in drawing 1, scheme 3 and 4. Reduction must be perfect in order for the wires to stabilize the humeral palette (**Fig. 12**). 

 4. When the wires are introduced in the distal fragment, the surgeon’s left index finger should be on the medial epicondyle.

 5. Each wire should be advanced slowly. When the tip of the wire becomes palpable under the skin, it must be withdrawn a few millimeters, not to create a source of irritation for the ulnar nerve.

 6. After the fracture is partly stabilized with the two wires in the ulnar column of the palette, two more wires are inserted, starting from the distal fragment as presented in **Drawing 1**, scheme 5, 6, 7 and 8, into the lateral column (**Fig. 13**).

**Drawing 1:**
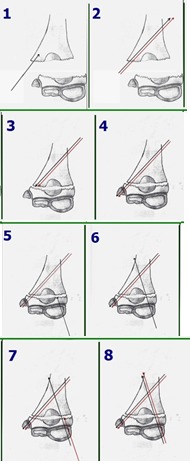
After the fracture is partly stabilized with the two wires in the ulnar column of the palette, two more wires are inserted, starting from the distal fragment

Option II: 

 The first wires are easier to place on the side of the pillar opposite to the olecranon fossa. This option supposes the insertion of a wire in each column, as presented in **Drawing 2**, scheme 1 and 2. To ease placement, a guide may be used after the first wires are introduced, as shown in **Drawing 2**, scheme 3. 

**Drawing 2:**
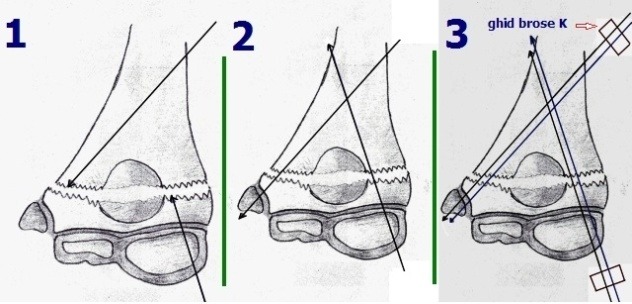
The first wires are easier to place on the side of the pillar opposite to the olecranon fossa. This option supposes the insertion of a wire in each column

 Option III: 

 Perform steps 1 and 2 from Option II.

 3. Before reduction, the wires for the lateral column are placed in the distal fragment, through the fractured surface and out through the condyle, as shown in **Drawing 3**, scheme 1.

4. The fracture is reduced and the four wires are driven in those in the medial column, into the medial epicondyle, and those in the condyle through the lateral column and into the medial cortex of the humerus, **Drawing 3**, scheme 2.

**Drawing 3:**
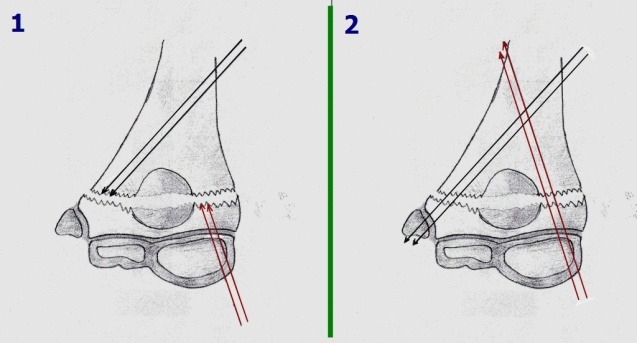
Before reduction, the wires for the lateral column are placed in the distal fragment, through the fractured surface and out through the condyle. The fracture is reduced and the four wires are driven in those in the medial column, into the medial epicondyle, and those in the condyle through the lateral column and into the medial cortex of the humerus

**Fig. 14:**
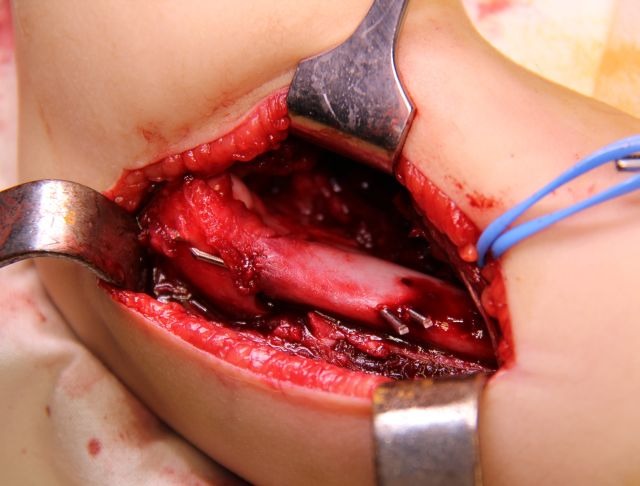
The wires are cut 5 mm from the cortex. The fracture must be anatomically reduced.

The wires are cut to a length that facilitates their removal later on (**Fig. 14**). The stability of the fixation is verified and the olecranon fossa is checked for obstructions. If the radial nerve has not been exposed, care must be taken not to injure it while closing the muscle layer of the wound. The wound is closed in an anatomical fashion. The wires may be removed at two weeks after surgery.

## Results

 The patients ages ranged 3 to12 years old, the mean age for girls was 7,3 years and 6,8 for boys. The hospitalization ranged 3 to 7 days, the average period being of 5 days. The wires had been pulled out after 21 days. The total recovery of the flexion and extension motion of the elbow was, depending on the age, between 21 and 40 days with an average period of 30 days. 

There were 5 cases of minor complications: in 3 cases the wires migrated outwards up to the 10th day and in 2 cases the wires were found in the olecranon fossa. The CT exam highlighted the impingement effect and the wire, which passed through the olecranon fossa, had to be removed between the 7th and the 9th day. There were no reported cases of cubitus varus or valgus.

 The "double X" wire fixation is a stable method of osteosynthesis, a fact that can be verified during surgery.

 The wires must be completely contained within the two columns of the olecranon fossa. If a wire should pass through the fossa, it would impinge on the olecranon and rehabilitation would not restore full extension in the expected 3–4 weeks. 

 Small comminuted fragments, invisible to imaging studies, must be removed in order to prevent aberrant callus formation, which may restrict elbow motion (**Fig. 18**).

 If the wires are placed correctly, rehabilitation may begin in the third day after surgery, by using first passive, then active motion. Two or three weeks after the surgery, the elbow motion should return to normal. 

 This type of internal fixation does not require subsequent immobilization.

**Fig. 15:**
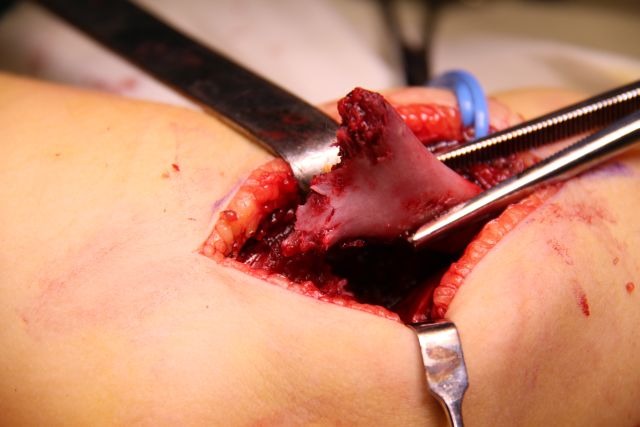
Comminuted fragments still attached to the proximal fragment, not apparent on X-ray.

## Discussions 

Supracondylar humeral fractures in children may be treated in many ways. The most used method is closed reduction and immobilization in a cast [**3,4**].

 Closed reduction is the easiest in the first six hours after the fracture [**6**] and yields the best results when performed under general anesthesia and if the reduction maneuvers match the displacement of the fracture: posterior/anterior varus/valgus and pronation/supination.

 Early reduction, general anesthesia, adequate maneuvers and a proper immobilization insure a favorable outcome for non-operatively treated cases. Consolidation and recovery of range of motion by orthopedic treatment is achieved in 92 % of the cases [**7**]. 

 Two wires may be applied in a "V" or "X" configuration in order to increase the stability of the fracture and to decrease the time spent in a cast. Other authors recommend and use fixation with three wires [**8-11**].

 A closed reduction and percutaneous pinning using the Judet technique was used for nonoperatively treated fractures with secondary displacement, unstable fractures and those with muscular interposition. Cast immobilization is required for 3–4 weeks.

**Fig. 16:**
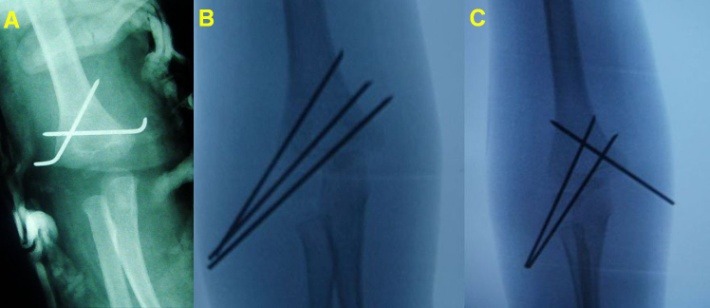
Other techniques for wire fixation: A. Crossed wires. B. San Antonio technique. C. San Diego technique

 A closed or open reduction followed by fixation with two crossed wires grants more stability, but also requires immobilization (**Fig. 16 A**).

 In the San Antonio (Wilkins) technique, three wires are inserted through the condyle (**Fig. 16 B**). The fixation is sturdier than the Judet or crossed wires techniques, but the risk of wire placement through the olecranon fossa requires immobilization and that rehabilitation must start only after wire removal. 

 The San Diego technique (**Fig. 16 C**) uses three wires, two inserted laterally through the condyle, fanning out medially, and a third through the medial epicondyle. When we applied this technique, the elbow was immobilized for three weeks, for safety, and rehabilitation also was started at three weeks, after wire removal.

 E. W. Edmond et al. treat supracondylar humerus fractures by percutaneous pinning, inserting three wires laterally and one medially. They have analyzed the incidence of ulnar neuropathy from the medial wire [**8,12**]. In a series of 187 cases, 4 had ulnar nerve damage, out of which only 2 were considered to be caused by the surgery [**12**].

 In our clinic, we used the four-wire "double X" internal fixation technique. In case of fractures with oblique line of fracture, it is more difficult to maintain a stable reduction without or with pinning by other techniques, and sometimes joint stiffness and/or ulnar nerve palsy appears. Burnei’s “double X" pinning ensures stability and avoids these potential complications.

 The wires’ diameters are chosen according to the age of the patient. Percutaneous pinning under image intensifier control was used for patients to whom closed reduction was possible and stable while the elbow was held in flexion by using a bandage. 

**Fig. 17:**
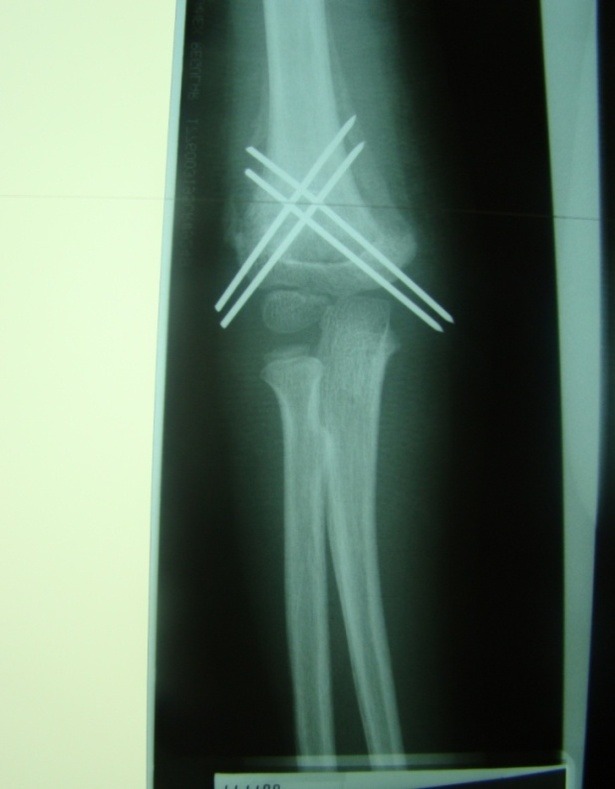
X-ray image of an hypertrophic callus formed after an old, neglected fracture was treated using the open technique.

 Our experience, consisting of 56 cases treated between 2001 and 2011 [**13**] allows us to state that, after this treatment method, children quickly regain elbow mobility. Old, neglected fractures operated by using this technique, exhibit hypertrophic callus after healing, but elbow motion is normal due to early mobilization and sturdy fixation (**Fig. 17**). Three days after surgery, the patients can actively move the elbow, grasp objects and, after 30 days, regain full range of motion. Five of our patients were able to take written tests 3–10 days after surgery. For patient comfort and for pain reduction, our patients wore an elbow brace for the first five days.

## Conclusions

 Double X" fixation for displaced supracondylar humerus fractures in children can be done in a minimally invasive fashion, by percutaneous pinning under the image intensifier control, or by open surgery. This technique has the advantage of strong stabilization of the fracture and the lack of subsequent immobilization. At 3–4 weeks after surgery, patients exhibit normal elbow motion after a cursory round of physical rehabilitation. 

 It is significant that patients may perform activities of daily living just three days after surgery and may even take a written exam.

 Acknowledgement

 This paper was co-financed by the Social European Found by the The Sectorial Operational Human Resources Developing Program 2007-2013, EXCEL-FIN Project, identification number of the contract: POSDRU/107/1.5/S/82839.
